# Incidence, risk factors and clinical outcomes of acute kidney injury associated with scrub typhus: a retrospective study of 510 consecutive patients in South Korea (2001–2013)

**DOI:** 10.1136/bmjopen-2016-013882

**Published:** 2017-03-15

**Authors:** Kyungo Hwang, Ha Nee Jang, Tae Won Lee, Hyun Seop Cho, Eunjin Bae, Se-Ho Chang, Dong Jun Park

**Affiliations:** 1 Department of Internal Medicine, Gyeongsang National University Hospital, Jinju, Korea; 2 Department of Internal Medicine, Changwon Gyeongsang National University Hospital, Changwon, Korea; 3 Department of Internal Medicine, Gyeongsang National University School of Medicine, Jinju, Korea; 4 Institute of Health Science, Gyeongsang National University, Jinju, Korea

**Keywords:** Scrub typhus, Acute Kidney Injury, RIFLE

## Abstract

**Objectives:**

Renal involvement in scrub typhus ranges from simple urinary abnormalities to acute kidney injury (AKI) leading to death. This study evaluated the incidence, predictors and prognosis of AKI associated with scrub typhus according to the RIFLE (risk, injury, failure, loss, end-stage kidney disease) criteria.

**Methods:**

We retrospectively evaluated the medical records of patients diagnosed with scrub typhus from January 2001 to November 2013 in Gyeongsang National University Hospital.

**Results:**

During the study period, 510 patients were diagnosed with scrub typhus and the incidence of AKI was 35.9%. There were 132 (25.9%) patients at risk, 37 (7.3%) with injury and 14 (2.7%) with failure. In comparison with the non-AKI group, the AKI group was older (73.9 vs 63.4 years, p<0.001) and had more comorbidities such as hypertension, diabetes mellitus and chronic kidney disease (CKD). AKI frequently occurs in hypertensive patients taking angiotensin receptor blockers or ACE inhibitors (p=0.002), and in patients with diabetes with higher glycated haemoglobin levels (p=0.033). Haematuria and proteinuria were more frequent in the AKI group. There was no relationship between the severity of proteinuria and occurrence of AKI. Intensive care unit admission and death were more frequent in the AKI group. The renal function of most patients with AKI recovered without sequelae, except for 1 patient who had underlying CKD. Multivariate analysis showed that age, presence of CKD, serum albumin level and time to hospital presentation after symptom onset were independent predictors of AKI in patients with scrub typhus.

**Conclusions:**

Our current results suggest that the presence of underlying CKD, older age, lower serum albumin level and time to hospital presentation after symptom onset were important risk factors to determine occurrence of AKI. Whether earlier diagnosis and treatment in patients with the above risk factors reduce the incidence and severity of AKI deserves to be investigated.

**Strengths and limitations of this study**

- This study is a large single-centre study, including all patients with scrub typhus over about 12 years. Above all, the same diagnostic criteria and identical laboratory conditions were applied to all patients.
- Despite many patients being enrolled in our study patients with chronic kidney disease was small (5.3%) and this might exert an important effect on statistical analysis.
- A major limitation of this study was its retrospective design. Since the research relied on medical records, it might be insufficient to find other possible risk factors of acute kidney injury (AKI).
- Above all things, urine volume, important criteria of ‘RIFLE’ (risk, injury, failure, loss, end-stage kidney disease) criteria, could not be included. This might limit the power of our statistics in revealing the incidence of AKI associated with scrub typhus.

## Introduction

Scrub typhus is a mite-borne infectious disease caused by the intracellular Gram-negative bacteria *Orientia tsutsugamushi*. It is an acute febrile illness with the characteristic findings of high fever, rash and generalised symptoms such as myalgia and headache. The disease is common in Southeast Asia, Australia, Japan, China and South Korea during the autumn season.^1–3^ The main reservoir is rodents, and their mites act as both the reservoir and the vector. Human infection occurs when the larvae of the thrombiculid mite infected with *O. tsutsugamushi* bite people and suck human tissue fluid.^4–6^ The clinical prognosis of scrub typhus varies from mild-to-severe courses. Although most of the patients have a benign course, some suffer serious complications such as rickettsial infections causing disseminated multiorgan vasculitis. According to the organ involved, patients could present with pneumonitis, meningitis, encephalitis, myocarditis, acute pulmonary oedema, pericarditis, hepatitis and even multiple organ failure.^7–9^ Respiratory distress and encephalitis are the principal cause of death in patients with severe disease.^10^

Acute kidney injury (AKI) is a major global health issue and its incidence is markedly rising^11^ and affects an estimated 13–18% of hospitalised patients,^12^ resulting in increasing hospital stay, healthcare costs, poor short-term and long-term outcomes,^13^ especially in patients with chronic kidney disease (CKD).^14^ In patients with infectious disease, especially those with sepsis, the incidence of AKI is reported to range from 5% to 51%.^15^ ^16^ Renal involvement is not uncommon in scrub typhus, and ranges from simple haematuria or proteinuria, 10–20% incidence of scrub typhus, to severe complications, including acute renal failure,^17–20^ nephrotic syndrome^21^ and end-stage renal disease leading to long-term haemodialysis.^22^ It is known that the incidence of AKI in scrub typhus ranges from 8% to 40% according to the classification criteria used.^17^ ^23–26^ The risk factors and prognosis of AKI associated with scrub typhus have been poorly studied.^26^ We have encountered poor prognosis and long hospital stay in patients with scrub typhus if AKI was accompanied, especially in patients with comorbidities such as diabetes mellitus (DM), hypertension and CKD. Therefore, we analysed the clinical and laboratory data of AKI in patients with scrub typhus.

The aim of the present study was to evaluate the incidence, risk factors and clinical outcomes of AKI according to the RIFLE (risk, injury, failure, loss, end-stage kidney disease) classification in a large series of patients with scrub typhus.

## Patients and methods

### Registries

This study enrolled 510 patients with scrub typhus who were admitted to Gyeongsang National University Hospital from January 2001 to November 2013. Their medical records were reviewed, including demographic data, clinical presentations, laboratory findings and clinical outcomes, to determine the incidence, risk factors and clinical outcomes of AKI associated with scrub typhus.

### Definitions

A diagnosis of scrub typhus was made when patients had a scab (eschar), acute febrile illness, skin rash, headache, muscle aches, lymph node swelling, hepatosplenomegaly and a high initial indirect immunofluorescent antibody (IFA) titre. If the initial titre was low, a fourfold increase in titre was considered significant. Proteinuria was categorised as trace, +, ++ or +++ using a urine dipstick test. Haematuria was defined as more than three red blood cells per high magnification field. In patients with underlying hypertension, the use of drugs that can affect renal function, such as ACE inhibitors (ACEi) or angiotensin receptor blockers (ARB) and diuretics, was investigated. In patients with DM, glycated haemoglobin was used to evaluate the degree of blood sugar control.

CKD was defined as an estimated glomerular filtration rate (eGFR) <60 mL/min/1.73 m^2^ using the Modification of Diet in Renal Disease study (MDRD) formula (1.86×Serum creatinine)**−**1.154×(age)**−**0.203)×(0.74 if female)×(1.210 if black). Shock was defined as a systolic blood pressure ≤90 mm Hg. The method of creatinine formation was Jaffe one. RIFLE classification was first reported in 2004, whereas AKIN classification, later or modified version of ‘RIFLE’ classification, was reported in 2007. We have usually used the RIFLE classification for AKI incidence in our institution because it can easily be applied when the baseline serum creatinine is known and has been largely validated in terms of determining the incidence of AKI. Furthermore, since we enrolled our patients from the year 2001, we used the original version for classification of AKI. AKI was defined and categorised according to the RIFLE classification.^27^ We used the serum creatinine level and eGFR to establish the RIFLE category because we had no data on the 6-hour and 12-hour urine volumes. If the baseline creatinine values were unknown, the serum creatinine was estimated using the MDRD formula and assuming eGFR=75 mL/min/1.73 m^2^ as the normal value. To evaluate the incidence, risk factors and clinical outcomes of AKI associated with scrub typhus, we divided the patients into AKI and non-AKI groups. To show the distribution of AKI cases, we annually evaluated the incidence of AKI associated with scrub typhus and compared it as an interquartile. On the basis of the occurrence of AKI, we also compared the clinical and laboratory characteristics of patients with CKD among those with scrub typhus in the AKI and non-AKI groups. We evaluated the severity of AKI categorised using RIFLE in the CKD versus non-CKD group.

### Statistical analysis

All measurements are the mean±SD. Pearson's χ^2^ and Fisher's exact tests were used to analyse qualitative differences. The parametric Student's t-test was used to compare the means of samples with similar variances. Multivariate logistic regression analysis was used to identify significant risk factors for the occurrence of AKI from among the risk factors identified in univariate analyses. The statistical analysis was performed using SPSS for Windows software (V.21.0; SPSS, Chicago, Illinois, USA). A p value <0.05 was taken to indicate statistical significance.

## Results

### Clinical and laboratory findings in all patients

Table 1 summarises the demographic data, underlying diseases, clinical symptoms and signs, and laboratory findings of the 510 patients diagnosed with scrub typhus: 62, 108, 76 and 264 patients in the first, second, third and fourth quartiles, respectively. The average patient age was 57.9 years and 48.0% were male. In total, 97 patients had underlying hypertension, of whom 22 took an ACEi or ARB (4.3%) and 12 took diuretics (2.4%). There were 61 patients with diabetes (12.0%) and 27 had CKD (5.3%). The average time to hospital presentation after symptom onset was 6.5 days.

**Table 1:** Clinical and laboratory data of the AKI group and non-AKI group

	Total (n=510)	AKI (n=183)	Non-AKI (n=327)	p Value
Age (year)	57.94±18.85	64.47±15.31	54.29±19.67	<0.001
Male	245 (48.0%)	97 (53.0%)	148 (45.3%)	0.097
HTN	97 (19.0%)	48 (26.2%)	49 (15.0%)	0.002
ARB use	22 (4.3%)	15 (8.2%)	7 (2.1%)	0.002
Diuretics use	12 (2.4%)	6 (3.3%)	6 (1.8%)	0.364
DM	61 (12.0%)	32 (17.5%)	29 (8.9%)	0.005
CKD	27 (5.3%)	19 (10.4%)	8 (2.4%)	<0.001
Time to hospital presentation after Symptoms onset	6.52±6.76	9.70±27.25	5.36±9.18	0.035
Symptoms and signs				
Fever	346 (85.0%)	128 (87.1%)	218 (83.8%)	0.392
Myalgia	102 (25.1%)	32 (21.8%)	70 (26.9%)	0.284
General weakness	48 (11.8%)	14 (9.5%)	34 (13.1%)	0.338
Eschar	200 (39.2%)	80 (43.7%)	120 (36.7%)	0.131
WCC (x10^3^/µL)	7.16±3.54	7.56±3.77	6.92±3.39	0.055
Haemoglobin (g/dL)	12.29±1.75	11.91±1.69	12.51±1.76	0.000
CRP (mg/L)	49.91±54.27	56.16±62.53	46.04±48.17	0.072
HbA1c (%)	7.59±2.35	8.10±2.42	6.87±2.07	0.033
Albumin (g/dL)	3.45±0.60	3.19±0.61	3.60±0.55	<0.001
AST (U/L)	112.7±400.6	150.9±647.2	90.7±104.0	0.213
ALT (U/L)	91.0±167.2	104.5±242.4	83.2±100.7	0.259
CK (IU/L)	278.25±673.77	371.05±897.71	192.68±345.53	0.082
Creatinine (mg/dL)	0.75±0.46	0.77±0.72	0.74±0.22	0.571
Haematuria	179 (36.5%)	80 (44.4%)	99 (31.8%)	0.005
Proteinuria	159 (32.3%)	73 (40.3%)	86 (27.7%)	0.005
Trace	100 (62.9%)	44 (60.3%)	56 (65.1%)	0.367
1+	43 (27.0%)	20 (27.4%)	23 (26.7%)	
2+	11 (6.9%)	6 (8.2%)	5 (5.8%)	
3+	5 (3.1%)	3 (4.1%)	2 (2.3%)	
Tsutsugamushi Ab titre	3234.04±5785.24	3762.6±3369.2	2862.2±6046.5	0.159
Hospital stay (days)	6.92±18.01	9.70±27.25	5.36±9.18	0.039
CRRT	3 (0.6%)	3 (1.6%)	0 (0.0%)	0.045
Shock	9 (1.8%)	5 (2.7%)	4 (1.2%)	0.293
Admission to ICU	8 (1.57%)	8 (4.4%)	0 (0.0%)	<0.001
Death	4 (0.8%)	4 (2.2%)	0 (0.0%)	0.016

Ab, antibody; AKI, acute kidney injury; ALT, alanine aminotransferase; ARB, angiotensin receptor blocker; AST, aspartate aminotransferase; CK, creatine kinase; CKD, chronic kidney disease; CRP, C reactive protein; CRRT, continuous renal replacement therapy; DM, diabetes mellitus; ESR, erythrocyte sedimentation rate; HbA1c, glycated haemoglobin; HTN, hypertension; ICU, intensive care unit; WCC, white cell count.

### Incidence of AKI and differences between the AKI and non-AKI groups

**Figure 1:**
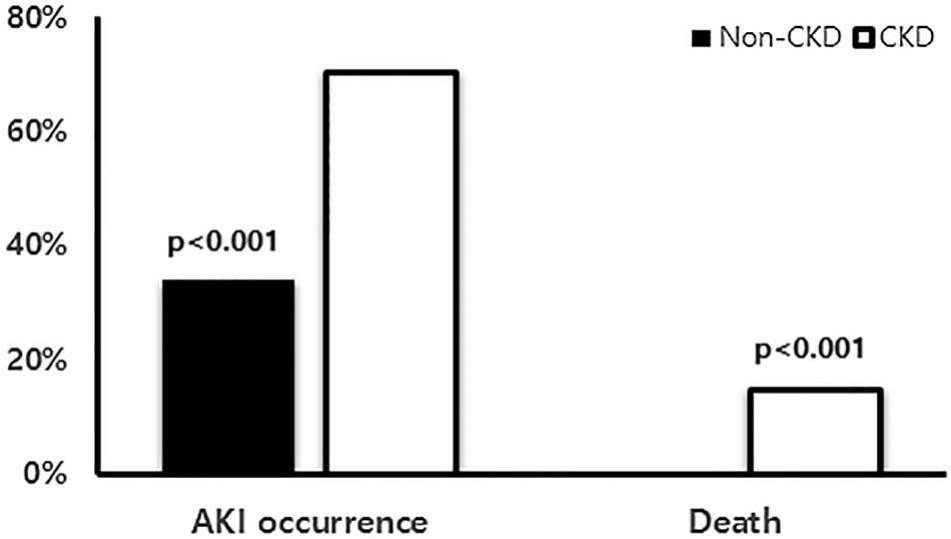
Percentage of AKI occurrence and death according to presence of CKD. AKI, acute kidney injury; CKD, chronic kidney disease.

**Figure 2:**
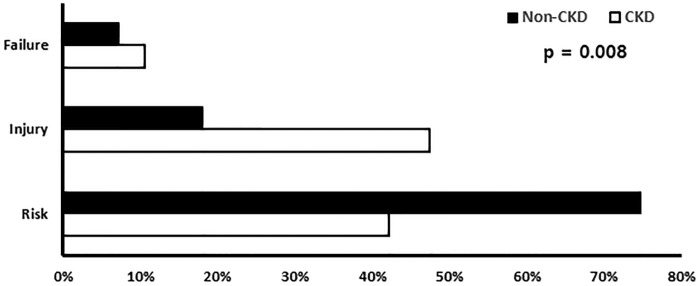
The comparison of AKI according to the RIFLE category in patients with CKD and non-CKD. AKI, acute kidney injury; CKD, chronic kidney disease; RIFLE, risk, injury, failure, loss, end-stage kidney disease.

AKI occurred in 183 of the patients (35.9%): 17 (27.4%), 38 (35.2%), 24 (31.6%) and 104 (39.4%) in the first, second, third and fourth quartiles, respectively (p=0.264). Of these, 132 patients were in the ‘risk’ category (25.9%), 37 were in the ‘injury’ category (7.3%) and 14 were in the ‘failure’ category (2.7%). There was no ‘loss’ or progression to end-stage renal disease. The AKI group was older (p<0.001). Hypertension, DM and CKD were more frequent in the AKI group (p=0.002, p=0.005 and p<0.001, respectively) compared with the non-AKI group. ARB or ACEi were used more frequently in patients with AKI with HT, whereas diuretics were not and glucose control was much poorer in the AKI group (p=0.033). The time to admission after symptom onset was longer in the AKI group (p=0.035). The serum albumin and haemoglobin levels were significantly lower in the AKI group (p<0.001 and p=0.000, respectively). Haematuria and proteinuria were more frequent in the AKI group (p=0.005 and p=0.005, respectively), although the degree of proteinuria did not differ between the two groups. The white cell count, C reactive protein, aspartate transaminase and alanine transaminase levels, and *O. tsutsugamushi* antibody titre did not differ between the AKI and non-AKI groups. In total, 27 patients (5.3%) had underlying CKD, of whom 19 (70.4%) developed AKI. This rate was significantly higher than that of patients without CKD (figure 1). Underlying renal function, measured using the MDRD formula, and the presence of haematuria were significant risk factors for AKI in these patients with CKD (table 2). The severity of AKI according to the RIFLE category was significantly greater in the patients with CKD compared with patients without CKD (figure 2). In univariate analysis, time to hospital presentation after symptom onset, older age, the presence of DM, HT and CKD, and lower albumin and haemoglobin levels were significant predictors of AKI. In the multivariate analysis, time to hospital presentation after symptom onset, older age, presence of CKD and lower albumin level (<3.5 g/dL) remained independent risk factors for AKI (table 3).

**Table 2:** Clinical and laboratory data of AKI and non-AKI with CKD

	Total (n=27)	AKI (n=19)	Non-AKI (n=8)	p Value
Age (year)	72.59±10.52	73.95±9.03	69.38±13.58	0.311
Male	14 (51.9%)	11 (57.9%)	3 (37.5%)	0.420
HTN	11 (40.7%)	10 (52.6%)	1 (12.5%)	0.090
ARB use	3 (11.1%)	3 (15.8%)	0 (0.0%)	0.532
Diuretics use	3 (11.1%)	3 (15.8%)	0 (0.0%)	0.532
DM	9 (33.3%)	7 (36.8%)	2 (25.0%)	0.676
Time to hospital presentation after symptom (day)	5.96±4.57	6.11±5.12	5.50±2.35	0.784
Symptoms and signs				
Fever	18 (75.0%)	12 (70.6%)	6 (85.7%)	0.629
Myalgia	10 (41.7%)	7 (41.2%)	3 (42.9%)	1.000
General weakness	3 (12.5%)	2 (11.8%)	1 (14.3%)	1.000
Eschar	16 (59.3%)	12 (63.2%)	4 (50.0%)	0.675
WCC (x10^3^/µL)	8.39±4.20	8.16±4.75	8.95±2.64	0.664
Haemoglobin (g/dL)	11.01±1.51	10.74±1.44	11.68±1.56	0.144
CRP (mg/L)	83.18±73.52	82.24±80.14	85.19±62.02	0.928
HbA1c (%)	7.99±1.79	7.87±1.90	8.80	0.664
Albumin (g/dL)	3.10±0.62	3.01±0.67	3.31±0.48	0.258
AST (U/L)	411.44±1659.31	557.21±1975.12	65.25±23.43	0.493
ALT (U/L)	189.67±567.68	244.47±674.10	59.50±31.46	0.450
eGFR (mL/min/1.73 m^2^)	49.42±8.02	47.15±8.28	53.95±5.42	0.048
Haematuria	16 (59.3%)	14 (73.7%)	2 (25.0%)	0.033
Proteinuria	15 (55.6%)	12 (63.2%)	3 (37.5%)	0.398
Trace	4 (26.7%)	3 (25.0%)	1 (33.3%)	0.891
1+	5 (33.3%)	4 (33.3%)	1 (33.3%)	
2+	4 (26.7%)	3 (25.0%)	1 (33.3%)	
3+	2 (13.3%)	2 (16.7%)	0 (0.0%)	
Tsutsugamushi Ab titre	2725.71±3087.79	3080.00±3463.41	1840.00±1798.13	0.420
Hospital stay (days)	6.59±6.80	6.63±7.67	6.50±4.53	0.964
CRRT	2 (7.4%)	2 (10.5%)	0 (0.0%)	0.567
Shock	3 (11.1%)	2 (10.5%)	1 (12.5%)	1.000
Admission to ICU	3 (11.1%)	3 (15.8%)	0 (0.0%)	0.532
Death	4 (14.8%)	4 (21.1%)	0 (0.0%)	0.285

Ab, antibody; AKI, acute kidney injury; ALT, alanine aminotransferase; ARB, angiotensin receptor blocker; AST, aspartate aminotransferase; CK, creatine kinase; CKD, chronic kidney disease; CRP, C reactive protein; CRRT, continuous renal replacement therapy; DM, diabetes mellitus; eGFR, estimated glomerular filtration rate by the Modification of Diet in Renal Disease study (MDRD); ESR, erythrocyte sedimentation rate; HbA1c, glycated haemoglobin; HTN, hypertension; ICU, intensive care unit; WBC, white cell count.

**Table 3:** Risk factors for the development of scrub typhus associated AKI

	Univariate analysis		Multivariate analysis	
Characteristics	p Value	OR (95% CI)	p Value	OR (95% CI)
Age (>65 years)	0.000	2.804 (1.931 to 4.073)	0.002	1.965 (1.270 to 3.040)
HTN	0.002	2.017 (1.289 to 3.157)	0.211	1.403 (0.825 to 2.386)
DM	0.005	2.178 (1.270 to 3.734)	0.188	1.516 (0.816 to 2.816)
CKD	0.000	2.808 (1.917 to 4.114)	0.013	3.526 (1.305 to 9.525)
Albumin (<3.5 g/dL)	0.000	2.226 (1.466 to 3.379)	0.001	2.095 (1.367 to 3.211)
Haemoglobin (<12 g/dL)	0.032	1.495 (1.035 to 2.160)	0.769	0.938 (0.613 to 1.437)
Time to hospital presentation after symptom onset (>7 day)	0.034	1.601 (1.039 to 2.465)	0.042	1.625 (1.017 to 2.597)

AKI, acute kidney injury; CKD, chronic kidney disease; DM, diabetes mellitus; HTN, hypertension.

### Outcomes of AKI

The hospital stay was much longer in the AKI group (p=0.039). The majority of surviving patients (99%) recovered from the AKI. Three patients underwent continuous renal replacement therapy (CRRT) in the AKI group, of whom two had underlying CKD. Eight patients required intensive care unit (ICU) treatment for AKI. Four (0.8%) patients with AKI died and all had underlying CKD: two underwent CRRT, while the other two did not. The death rate was also higher in patients with CKD, compared with patients without CKD (figure 1). Renal function did not recover fully in only 1 (7.7%) of the surviving patients with CKD, whereas it recovered in all of the patients without CKD, including 1 who underwent CRRT. There was no information for five patients in the non-CKD group due to early discharge and follow-up loss. However, the renal function of these patients most likely recovered because all initially had mild AKI (two ‘risk’ patients and three ‘injury’ patients). There was no case of end-stage renal disease requiring maintenance haemodialysis after the 3-month follow-up. The major cause of death was uncontrolled infection.

## Discussion

In our series, the incidence of AKI in scrub typhus was 35.9%. According to the RIFLE classification, AKI was associated with ‘risk’ (25.9%), ‘injury’ (7.3%) and ‘failure’ (2.7%). AKI was more frequent in older patients who had lower serum albumin level and visited hospital late after symptom onset and with underlying disease, such as uncontrolled DM or HT, taking ACEi or ARB. In patients with underlying CKD, AKI was more frequent and had a poorer prognosis in terms of severity and survival.

There are few studies on the clinical outcome of AKI according to the RIFLE classification associated with scrub typhus.^17^ ^25^ Basu *et al*^25^ first reported that the RIFLE classification is a valid indicator of AKI in acute febrile diseases, including scrub typhus, and can predict renal replacement therapy and the risk of mortality. Compared with our study, the incidence of AKI associated with scrub typhus was higher (42.6% vs 35.9%) and the severity of AKI was greater: ‘risk’ (20.2% vs 25.9%), ‘injury’ (11.2% vs 7.3%) and ‘failure’ (11.2% vs 2.7%). In addition, 5.9% of their patients underwent dialysis, the risk of mortality was associated with AKI severity, and mortality was significantly higher, at 13.3%, compared with our study. Our study also showed that all the patients who died had AKI. Basu *et al* focused on the relationship between febrile infectious diseases and AKI according to the RIFLE classification, including other acute febrile diseases, and not the relationship between AKI and scrub typhus itself or predictors of AKI.

Attur *et al*^17^ demonstrated that thrombocytopaenia and ICU treatment were predictors of acute renal damage in patients with scrub typhus and AKI occurred in 23.2% of their patients. The renal injury classifications were as follows: ‘risk’ (8.9%), ‘injury’ (5.0%) and ‘failure’ (10.8%); and replacement therapy was given to 10% of their patients. The lower incidence of AKI (23.2% vs 35.9%) compared with our study might be explained by the difference in the age of the enrolled patients (40 vs 58 years). Age was an important predictor of AKI in our study. In addition, patients with underlying diseases such as CKD, DM and hypertension were included in our study. The underlying disease tends to increase in frequency with age. Easy access to a tertiary hospital might reduce the incidence and severity of AKI because of the regional characteristics of small areas. Our study also showed that the time from symptom onset to hospitalisation was associated with AKI incidence.

The mechanism of AKI in scrub typhus is unclear. One plausible theory is that the invasion of *Rickettsia* induces vasculitis, leading to direct renal involvement.^18^ ^19^ However, renal biopsies have not revealed evidence of renal vasculitis associated with scrub typhus but have shown inflammation and proliferation of the glomerular tubule interstitium^4^ ^5^ and tubular necrosis due to direct involvement of tubules by *Rickettsia*.^17–19^ Membranous nephropathy has also been demonstrated,^21^ although we cannot completely rule out renal vasculitis because very few renal biopsies have been performed in scrub typhus.

Dumler *et al*^28^ suggested that a decrease in renal blood flow accompanied by extravasation resulting from systemic vasculitis is the cause of pre-renal AKI. Hypoalbuminaemia caused by the leakage of serum albumin due to vasculitis is also postulated to be associated with AKI.^20^ Although hypoalbuminaemia was not a predictor of acute renal injury in previous studies,^17^ ^25^ the serum albumin level was significantly lower in our patients with AKI, and serves as an important predictor of AKI. Hypoalbuminaemia is also an important marker of severe infection and/or byproduct of chronic disease such as diabetes, hypertension and CKD. Others have suggested different mechanisms, such as pan-vascular coagulation^17^ ^19^ ^20^ and rhabdomyolysis,^17^ ^19^ but were not able to prove these hypotheses. The creatine kinase level did not differ between the two groups in our study.

A close relationship between AKI and mortality has been reported. The mortality from scrub typhus in endemic areas in India is 2–12.2%.^23^ ^24^ The mortality rates in two studies of AKI associated with scrub typhus were 13.3% and 0.8%.^17^ ^25^ All of the deaths occurred in the ‘failure’ category of AKI. In our series of 510 patients, 4 died (0.8%) and all were in the ‘failure’ category of AKI. Our patients had underlying CKD. Previous reports did not identify CKD as a risk factor for AKI or mortality.^17^ ^23–25^ The prognosis of AKI associated with scrub typhus has rarely been reported.^17^ ^25^ The renal prognosis after AKI is good if the patient survives. Permanent dialysis treatment was needed in one case study.^22^ In our series, only one case did not recover to baseline renal function and there was no permanent renal loss requiring long-term dialysis.

The main methods in scrub typhus diagnostics remain serology, but currently available serological tests have limitations. Of that, the gold standard for scrub typhus is IFA despite some limitations. Serological tests are most reliable when a fourfold rise in antibody titre is shown.^29^ If the patient lives in a non-endemic area, the diagnosis can be made from a single acute serum sample to require a cut-off antibody titre. This is impossible in patients living in endemic areas because antibodies can be found in up to 18% of healthy populations.^30^

A major limitation of this study was its retrospective design. In addition, since the research relied on medical records, the capacity to find other possible causes of AKI was limited and urine volume, an important factor in the RIFLE classification, could not be analysed. Therefore, the incidence of AKI might have been underestimated. However, we believe that these limitations are overcome by the large participant pool and application of many variables to the statistical analysis. It was also a single-centre study, so relatively similar laboratory values applied, most patients were followed at the same facility, and the same diagnostic criteria and treatment were used.

Owing to global warming and an increase in travel to other countries, the incidence of contagious febrile diseases is on the rise both in developing and developed countries. Risk factors of AKI associated with both endemic and epidemic acute febrile illnesses such as malaria, leptospirosis, dengue fever, severe acute respiratory syndrome, Middle East respiratory syndrome and severe fever with thrombocytopaenia syndrome remain to be established. This study enrols the large patients and shows the several clinical and biochemical risk factors of AKI associated with scrub typhus. Therefore, as independent risk factors, time to hospital presentation after symptom onset, older age, presence of CKD and lower albumin level to predict AKI in our results can also be applied to predict AKI in the above febrile diseases. The high frequency and poor prognosis of AKI in patients with CKD among our results should also be kept in mind to evaluate AKI of above acute febrile diseases.

## Conclusions

AKI incidence associated with scrub typhus is 35.9%. Our current results suggest that the presence of underlying CKD, older age, lower serum albumin level and time to hospital presentation after symptom onset were important risk factors to determine occurrence of AKI. Whether earlier diagnosis and treatment in patients with the above risk factors reduce the incidence and severity of AKI deserves to be investigated.
